# Precursor Mediated Synthesis of Nanostructured Silicas: From Precursor-Surfactant Ion Pairs to Structured Materials

**DOI:** 10.3390/ma7042978

**Published:** 2014-04-11

**Authors:** Peter Hesemann, Thy Phung Nguyen, Samir El Hankari

**Affiliations:** 1 Institut Charles Gerhardt de Montpellier, UMR 5253, Université Montpellier 2, CC 1701, Place Eugène Bataillon, 34095 Montpellier cedex 05, France; E-Mail: phuong@vpscience.com; 2 School of Chemistry, University of Southampton, Highfield, Southampton SO17 1BJ, UK; E-Mail: s.el-hankari@soton.ac.uk

**Keywords:** template directed synthesis, anionic surfactant, nanostructured silica, silica hybrid materials

## Abstract

The synthesis of nanostructured anionic-surfactant-templated mesoporous silica (AMS) recently appeared as a new strategy for the formation of nanostructured silica based materials. This method is based on the use of anionic surfactants together with a co-structure-directing agent (CSDA), mostly a silylated ammonium precursor. The presence of this CSDA is necessary in order to create ionic interactions between template and silica forming phases and to ensure sufficient affinity between the two phases. This synthetic strategy was for the first time applied in view of the synthesis of surface functionalized silica bearing ammonium groups and was then extended on the formation of materials functionalized with anionic carboxylate and bifunctional amine-carboxylate groups. In the field of silica hybrid materials, the “anionic templating” strategy has recently been applied for the synthesis of silica hybrid materials from cationic precursors. Starting from di- or oligosilylated imidazolium and ammonium precursors, only template directed hydrolysis-polycondensation reactions involving complementary anionic surfactants allowed accessing structured ionosilica hybrid materials. The mechanistic particularity of this approach resides in the formation of precursor-surfactant ion pairs in the hydrolysis-polycondensation mixture. This review gives a systematic overview over the various types of materials accessed from this cooperative ionic templating approach and highlights the high potential of this original strategy for the formation of nanostructured silica based materials which appears as a complementary strategy to conventional soft templating approaches.

## 1. Introduction

Silica based materials with regular architectures on a mesoscopic length scale (2–50 nm) attracted great scientific interest due to their high potential for applications in the fields of catalysis, separation, sensing drug delivery, *etc*. These materials are characterized by high surface area, tunable pore size and well defined mesophase in terms of regular pore architecture. Since the discovery of soft templating approaches to mesoporous nanostructured silica in the early 1990s by Kuroda *et al*. [1–3] and scientists of the Mobil company, a great number of mesoporous silica materials featuring various architectures such as M41S, SBA, HMS or KIT materials have been introduced [4]. In a general way, the formation of these materials involve supramolecular cooperative self-assembly of cationic or non-ionic surfactants which act as structure directing agents (SDA). These lyotropic phases are used as soft template which allows casting the silica material during the hydrolysis-polycondensation reaction. This template directed hydrolysis-polycondensation process therefore allows accessing various types of structured silica materials resulting from the structure of the lyotropic phase of the SDA in the hydrolysis-polycondensation mixture. The surfactant organization in aqueous media plays a crucial role in the formation of siliceous solids exhibiting defined architectures. The supramolecular aggregation of surfactants depends on various parameters such as the length of the alkyl tails, the charge of the polar head group or the molecular shape of the surfactant. All these parameters directly affect the packing parameter *g* of the surfactants. In this respect, templating routes involving different types of soft templates allow controlling the mesophase formation and give rise to the formation of nanostructured silica mesophases with well defined architectures on a mesoscopic level such as 2*d*/3*d* hexagonal, cubic or lamellar architectures.

**Figure 1.**
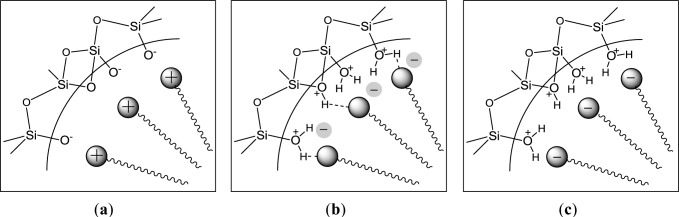
Precursor-surfactant interactions in the syntheses of mesoporous nanostructured silicas via template directed approaches in the presence of cationic surfactants (**a**); non-ionic surfactants (**b**); and anionic surfactants (**c**). Reprinted with permission from [9]. Copyright 2006 Wiley-VCH.

On a molecular level, strong interactions between SDA and precursor are the reason for the formation of highly structured silica mesophases. The nature of the used surfactant in combination with the reaction conditions was found to be essential in view of the formation of nanostructured phases. High affinities between soft template and silica forming phase are formed either via ionic interactions or hydrogen bonding. Figure 1 schematically illustrates the principal templating modes in the presence of cationic, anionic and non-ionic structure directing agents. In the case of the utilization of cationic surfactants, ionic interactions between the positively charged head groups of the surfactant and anionic silanolate groups are the cause of a strong affinity between these two phases. This classical S^+^I^−^ mechanism, encountered in the syntheses of M41S type materials such as MCM-41, involves hydrolysis-polycondensation reactions in alkaline reaction media (Figure 1a). On the other side, the interactions between the silanol groups and non-ionic templates (Figure 1b) are amplified in acidic reaction media due to the formation of hydrogen bonds, for example in the synthesis of SBA type materials [5–7]. Formally, the use of anionic surfactant should also be a promising method for the formation of nanostructured silica in acidic reaction media as partially protonated silanol species should be able to interact with negatively charged head-groups of anionic surfactant (Figure 1c). However, the formation of structured silica mesophases in the presence of anionic surfactants has never been observed under these conditions [8]. Hydrolysis-polycondensation of silica precursor in the presence of anionic templates always gives rise to the formation of amorphous silica phases, highlighting only weak affinity between anionic templates and the silica precursor and its hydrolyzed derivatives.

Due to their surface properties (high surface area and pore volume, *etc.*), their high chemical stability and their particular physical and physico-chemical properties, mesoporous nanostructured silicas were rapidly recognized as attractive heterogeneous support for various functional groups, for instance for luminescent, metal coordinating or chiral entities. The functionalization of the internal surface with functional groups thus opened the route towards a huge variety of functional silica based materials which found applications in the areas of separation, catalysis, sensing, drug delivery, *etc*. [9,10].

The functionalization of nanostructured silica phases can be achieved following three pathways [9]: (i) the subsequent modification by organic groups through the reaction of organosilanes with free silanol groups located on the surface of the pure mesoporous silica (*post-grafting*); (ii) the simultaneous co-condensation of organosilane and silica sours in a “one-pot” process (*co-condensation*); and finally the synthesis of periodic mesoporous organosilicas via hydrolysis-polycondensation reactions starting from di-or oligosilylated functional precursors. This last method should be considered separately, as it affords silica hybrid materials. In these polysilsesquioxanes, the organic functions are integrated within the scaffold of the solid. In contrast, post-synthesis grafting and co-condensation methods lead to the formation of surface functionalized silicas.

Following the initial studies of synthesis of nanostructured silica phases, the chemical functionalization via co-condensation reactions of silica based materials was rapidly investigated [11–14]. A general drawback of this method quickly appeared as the presence of the organic precursor in the hydrolysis-polycondensation mixture showed an unfavourable effect towards the formation of structured phases. As the micellar arrangement of the template in aqueous phase was hampered in the presence of organic precursors, the maximum quantity of organic precursor was generally found to be 20 mol%. Beyond this limit, only unstructured materials were obtained. However, in some special cases, structured phases were obtained from TEOS/organosilane molar ratios as high as 46/54 [15].

These studies clearly show that the formation of nanostructured silica phases depend on two different parameters:

(1) The formation of micellar phases via auto-assembly of surfactants. These phases act as soft templates for the formation of structured silica mesophases;
(2) A sufficient affinity of the silica forming phase towards the soft template in order to create supramolecular silica-surfactant aggregates and, *in fine*, nanostructured silica-surfactant nanocomposites. This affinity seems to be insufficient in the case of the use of anionic SDAs and therefore does not allow the formation of structured silica phases.

More recently, a new method was introduced for the formation of nanostructured silica and silica hybrid mesophases. This method is based on an amplification of the interactions between the soft template phase and the silica forming phase via the addition of ionic silylated precursors to the hydrolysis-polycondensation mixture. This strategy implies the formation of precursor-SDA ion pairs and therefore directly involves a contribution of the organic precursor in view of the generation of structured mesophases. This technique makes also possible the utilization of anionic surfactant as structure directing agents.

The aim of this article is to summarize the work in the area of precursor induced generation of silica based mesophases and to emphasize the particularities of this approach compared to the traditional soft templating approaches involving cationic and non-ionic SDAs. It will be shown that this “precursor mediated templating route” opens a highly universal and flexible access to functional and nanostructured silica based materials and should be considered as a complementary method to the classical templating strategies involving cationic and non-ionic SDAs.

### 2. Nanostructured Silicas via *“Ionic Templating”* from Precursor Surfactant Ion Pairs

Due to the low affinity of hydrolyzed silica precursors towards micellar aggregates of anionic surfactants, hydrolysis-polycondensation reactions in the presence of anionic SDAs always led to amorphous silicas. However, the use of anionic structure directing agents is highly desirable due to the commercial availability of a large variety of anionic surfactants and their rather low price. For these reasons, the use of anionic structure directing agents as templates for the formation of nanostructured silica phases was intensively studied.

**Figure 2.**
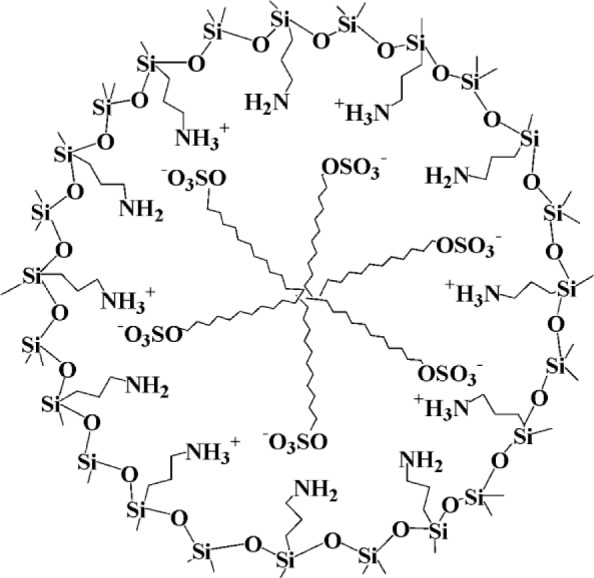
Ionic interactions between anionic surfactant and cationic co-structure directing agent (CSDA) in the synthesis of anionic surfactant template mesoporous silica (AMS). Reprinted with permission from [16]. Copyright 2003 American Chemical Society.

In 2003, Tatsumi *et al*. [16] reported that moderately structured silica phases are formed upon hydrolysis-co-condensation reactions of tetraethyl-*ortho*-silicate (TEOS) and 3-aminopropyl-triethoxysilane in the presence of anionic surfactants such as Sodium-dodecyl sulphate (SDS). In this synthesis of anionic-surfactant-templated mesoporous silica (AMS), the authors postulated the formation of structured silica mesophases due to increased electrostatic interactions between the micelles of the anionic surfactant and the *in situ* formed ammonium species (Figure 2). In this context, the presence of the silylated ammonium precursor appears to be essential for the formation of structured solids. These compounds are therefore considered as co-structure directing agents (CSDA), as they increase affinity of the silica mesophase towards the anionic micelle.

This synthetic strategy for the synthesis of these structured silica mesophases is of high interest for several reasons. Besides the fact that it enables the use of cheaper anionic surfactants in template directed hydrolysis-polycondensation processes to structured silica mesophases, this behaviour is of particular interest form a fundamental point of view as it illustrates that molecular interactions are responsible for the formation of structured phases. It therefore allows getting detailed comprehension of the formation mechanism of nanostructured silica phases in general. Additionally, it allows the direct formation of highly functionalized and well-structured silica mesophases as a result of ionic interaction between SDA and silylated precursor and provided a valuable route to materials with very high accessibility of functional groups. The quantity of accessible ammonium groups within these AMS materials was unexpectedly high with 3.4 mmol·g^−1^. This result was attributed to a “guiding” of the cationic ammonium groups via direct electrostatic interactions with the anionic structure directing agents together with the amine precursors, which therefore act as SDA-CSDA ion pairs.

This is therefore the first example of precursor-mediated generation of nanostructured silica phases. Contrarily to the formation of nanostructured silica mesophases in the presence of cationic or non-ionic surfactants, the formation of nanostructured phases is precursor-induced and not governed by siloxy-surfactant interactions. The route to anionic surfactant templated silica therefore does not only provide an alternative strategy for the formation of nanostructured silica materials but also allows accessing functional materials with very good accessibility of the immobilized functional groups due to a supramolecular templating. Very interestingly, the formation of supramolecular surfactant-precursor aggregates induces considerably accelerated reaction rates in hydrolysis-polycondensation reactions and led to a very rapid formation of silica material compared to similar reactions without added CSDA, thus emphasizing the importance of supramolecular aggregation for reaction kinetics.

Finally, the formation of pure nanostructured silica phases was achieved by calcination of the as-synthesized material via the thermal decomposition of both surfactant and chemically grafted aminopropyl groups.

This first work on AMS silica clearly shows that “anionic templating method” is a versatile strategy for the control of morphology and texture of siliceous materials. In the following, Che *et al*. [17–19] studied the synthesis of AMS materials in detail. In this work, long chain substituted carboxylates were used as structure directing agents. Contrarily to the formerly used sulphate surfactant, the degree of ionization of carboxylates/carboxylic acids can easily be controlled via the acidity/basicity of the reaction mixture since carboxylates are weak bases and are protonated and transformed to the corresponding carboxylic acids below pH 4–5. Together with X-ray diffraction, high resolution transmission electron microscopy was particularly used to characterize the formed silica mesophases on a mesoscopic length scale.

**Figure 3.**
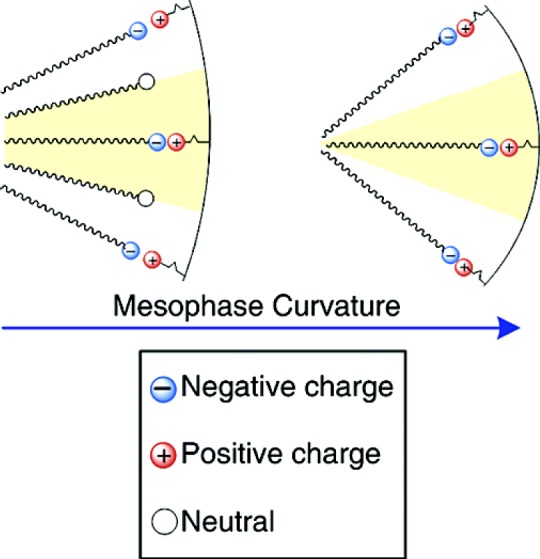
Control of the mesophase curvature in AMS mesophase formation via the surfactant ionization degree. Reprinted with permission from [18]. Copyright 2006 American Chemical Society.

**Figure 4.**
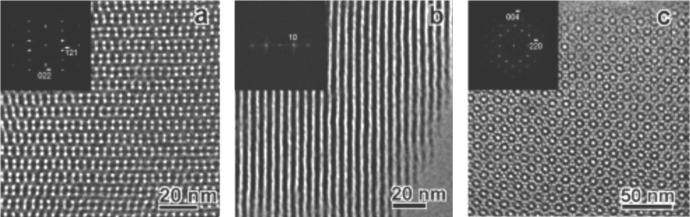
TEM-images of three AMS mesophases obtained under different reaction conditions. (**a**) acidic conditions: bicontinuous cubic $Ia\bar{3}d$ space group mesophase; (**b**) 2*d* hexagonal *p*6*mm* mesophase; (**c**) cage type cubic $Fd\bar{3}m$ space group. Reprinted with permission from [18]. Copyright 2006 American Chemical Society.

Mechanistic studies clearly showed that the anionic templating route allows controlling the type of formed silica mesophase in particular from the applied reaction conditions [18]. A clear relationship was found between the type of formed mesophase and the surfactant packing parameter *g* [20] of the anionic surfactant. As this latter factor depends on various other parameters such as the degree of ionization of the carboxylate surfactants, but also the charge density of the micelle or the nature of the CSDA (Figure 3), this approach provides a facile access to a variety of silica mesophases with defined architectures. For example, the architecture of AMS mesophases can be controlled from the acidity/basicity of the hydrolysis-polycondensation mixture or the quantity of the CSDA added to the reaction mixture. Figure 4 shows TEM-images of three samples obtained under acidic, neutral and basic reaction conditions. As a consequence of the pH induced change of the micellar arrangements, cubic mesophases displaying different space groups were formed under acidic and basic reaction conditions, whereas a material with 2*d* hexagonal architecture was formed in neutral reaction medium. These results are further supported via XRD of the obtained materials after calcination.

In this example, the observed changes in the structure of the formed mesophases can particularly be attributed to the degree of ionization of the surfactant, which is very sensitive to the acidity or basicity of the synthesis system. However, several other parameters also affect the architecture of the materials obtained by this combined SDA-CSDA approach. The surfactant packing has shown to be strongly affected by the following parameters:

- the geometrical size and the nature of the anionic head group of the surfactant;
- the length of the hydrophobic alkyl chain of the surfactant;
- the nature and geometrical size of the CSDA, and finally;
- the SDA/CSDA ratio in the hydrolysis-polycondensation mixture.

In this context, it has been shown that the nature and the constitution of the surfactant has a particular influence on the architecture of the formed materials [19]. Both the nature of the anionic head group (carboxylate, sulfonate, sulphate, phosphate) and the size and chain length of the alkyl tail result in mesophase changing via the modification of the organic/inorganic interface curvature. Thus, the molecular design of both surfactant and co-structure directing agent opened the route to a variety of differently structured materials.

Additionally, it was found that the experimental set-up has also deep influence on the architecture of the formed materials. In particular, it was observed that the architecture of the material can be controlled via the delay between the addition of TEOS to the hydrolysis-polycondensation mixture containing the surfactant and the CSDA [17]. In this way, materials with different types of cubic mesophases were obtained. All these results indicate that the anionic templating method is a highly flexible strategy for the formation of a large variety of silica mesophases with well-defined pore connectivities.

To summarize, the mesophase formation of AMS materials can easily be controlled and modified by a multitude of parameters. This approach is of particular interest as it appears as a highly versatile route to a large variety of well-defined mesoporous silicas displaying various types of mesophases [21]. Most of the syntheses reported here involve the use of carboxylate or sulphate surfactants. However, other types of anionic soft templates were also successfully employed for the synthesis of nanostructured silica phases following the SDA-CSDA approach. For example, amine-functionalized mesoporous silica nanoparticles (MSN) were obtained using a long chain substituted phosphate monoester [22]. Due to the low cytotoxicity of this surfactant, the formed mesoporous silica nanoparticles showed excellent biocompatibility and were successfully used as drug delivery carriers for hydrophobic drugs.

**Figure 5.**
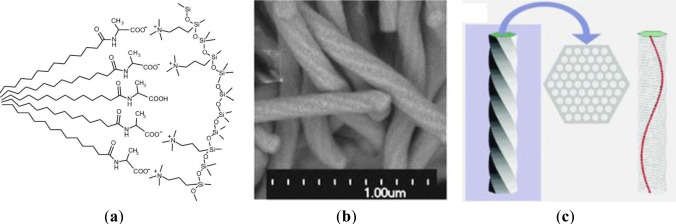
Formation of AMS mesophases with inherent chirality using chiral anionic surfactant; (**a**) Supramolecular precursor surfactant arrangement; (**b**) SEM image of chiral mesoporous silica; (**c**) Schematic drawing of a structural model of the chiral mesoporous material for TEM image simulation; cross-section of the material; schematic drawing of a single chiral channel in the material. Reprinted with permission from [23]. Copyright 2004 Nature Publishing Group.

The great potential of the anionic templating approach clearly appears in the area of the formation of mesoporous silicas with inherent chirality [23–25]. The use of chiral carboxylate surfactants, derived from natural amino acids, allowed the formation of materials displaying twisted hexagonal rod-like morphology. By controlling the ionic interactions between the anionic head group of the chiral anionic surfactant *N*-miristoyl-l-alanine sodium salt and the silica forming phase, a helicoidal silica mesophase is formed via a co-condensation reaction of a cationic CSDA with TEOS (Figure 5). In this way, twisted silica rods with approx. 150 nm of diameter and several μm in length are shaped. From TEM images of this material, the helical pitch along the rod can be estimated to be ~1.5 μm. It should be mentioned that the cross-section of the material shows a highly ordered silica mesophase with hexagonal *p6mm* architecture. The found values for pore diameter of 2.2 nm and *d*-spacing of 3.8 nm are in line with formerly found values of related non-chiral samples. These results were also confirmed by XRD measurements. The diffractograms of the obtained chiral material nicely showed the 1 0, 1 1 and 2 0 reflexions characteristic for materials with 2*d* hexagonal architecture. Detailed studies on the formation of these chiral phases showed that the synthesis of this type of materials is very delicate and depends on multiple parameters such as the stirring rate of the hydrolysis-polycondensation mixture [24]. The exact control of the reaction conditions is therefore essential for the reproducibility of the reactions and the formation of materials with inherent helicity.

**Figure 6.**
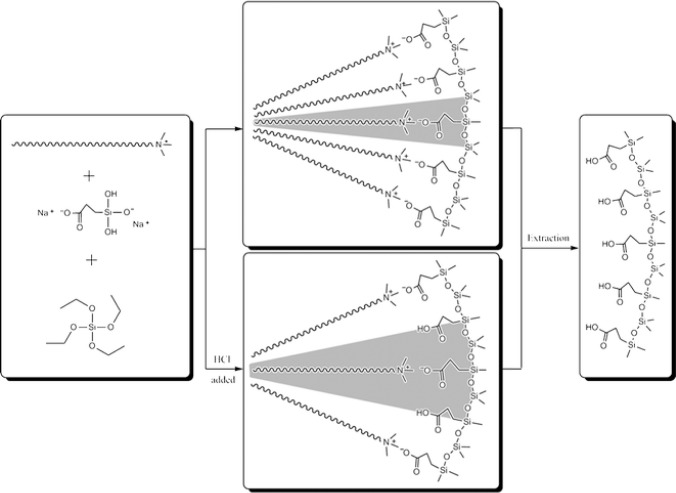
Formation of carboxylate functionalized nanostructured silica from cationic SDA/anionic CSDA ion pairs. Reprinted with permission from [26]. Copyright 2007 Royal Society of Chemistry.

Following the results concerning the synthesis of anionic surfactant _emplate mesoporous silica which led to the formation of surface functionalized silicas containing amine and ammonium groups, a similar strategy was applied on the synthesis of carboxylic group functionalized mesoporous silicas. The formation of this type of material involves inverse ion pairs with respect to the synthesis of AMS mesophases, *i.e.*, anionic co-structure directing agent and cationic surfactant (Figure 6) [26,27]. As discussed before, the charge density of the carboxylate function has large influence on the architecture of the formed materials and therefore provides an efficient control concerning the textural properties of the formed material. By controlling the acidity and thus the degree of ionization of the carboxylate groups, different interactions between the surfactant phase and the CSDA are created, which allow controlling the interphase curvature and finally the architecture of the formed mesophase. This approach was also used for the synthesis of MCM-41 type mesoporous silica nanospheres with high degree of accessible organic function located on the surface of the material [28]. Interestingly, this approach is not limited on the use of cationic structure directing agents and the formation of MCM-41 type materials. The use of the non-ionic surfactant Pluronic P123 allows accessing SBA-15 type materials with pore diameters in the range from 4 to 7 nm [29].

**Figure 7.**
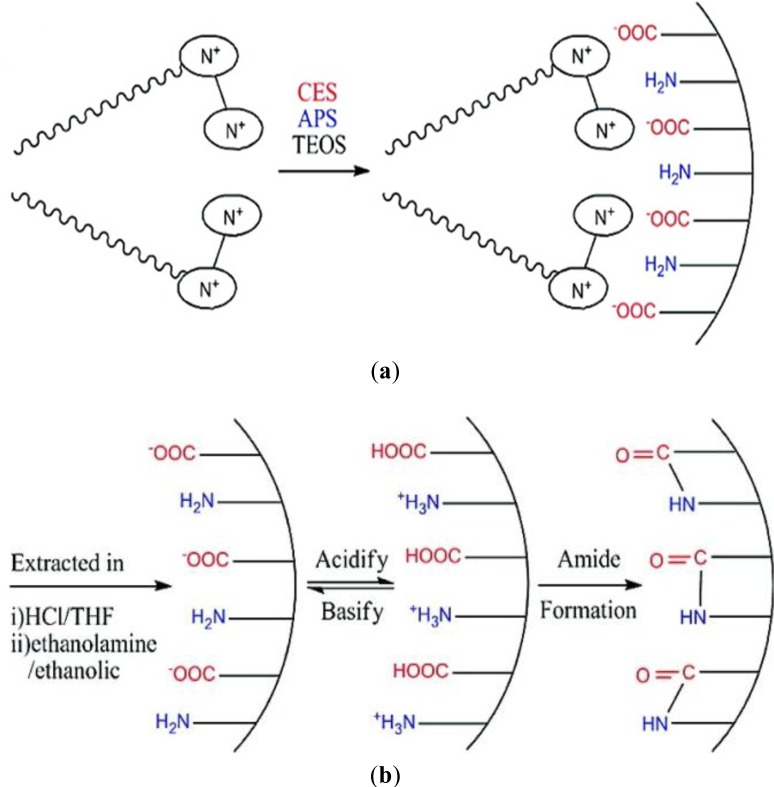
Schematic illustration of the interactions between cationic Gemini surfactant and the CSDAs (APS and CES) and switching process between the base, acid and amide formation; (**a**) Schematic illustration of the interactions between the Gemini surfactant and the co-structure directing agents; (**b**) Switching process between acid and base; amide formation. Reprinted with permission from [30]. Copyright 2007 American Chemical Society.

Finally, amphoteric amino acid bifunctional mesoporous silica materials were synthesized via a combined templating approach [30]. Functionalized silicas bearing both amine/ammonium and carboxylate/carboxylic acid functions were synthesized via hydrolysis-polycondensation reactions involving simultaneously amine and carboxylate precursors (Figure 7). The reactions were carried out in the presence of cationic, anionic and mixed cationic/anionic surfactants. The best results were obtained for hydrolysis-polycondensation reactions in the presence of cationic Gemini surfactant, which led to the formation of a organized cubic silica mesophase [30] probably due to a synergy between the classical structuration induced by the silica forming phase and ionic contribution from the anionic precursor/cationic surfactant ion pairs. Interestingly, the formed material behaves as an amino acid with an isoelectic point close to that of a free amino acid and amphoteric behaviour with allows switching from a cationic to a fully anionic surface. Furthermore, the formation of amide groups illustrates the carboxylate and amine groups are located closely together on the surface of the material (Figure 7).

In conclusion, various types of ionically modified surface functionalized silicas displaying high structural regularities are accessible via the co-structure directing agent method. This method involves the use of ionic trialkoxysilylated precursor compounds which allow increasing the affinity towards the surfactant phase via the formation of ionic interactions. The strategy was applied on the formation of surface functionalized silicas bearing both cationic and anionic groups, but allowed also accessing nanostructured bifunctional amphoteric materials containing both amine/ammonium and carboxylate/carboxylic acid functions. The technique is generally applicable and allows accessing a large variety of structured materials depending on a judicious choice of the SDA/CSDA ion pairs together with the meticulous control of the reaction conditions. Furthermore, this approach led to functionalized silica materials bearing different types of ionic groups. These functional groups are located on the surface of the materials due to a directing effect during the materials’ formation.

## 3. Ionosilica Hybrid Materials

The work of Che *et al*. [17–19,21,23,24,26,27] beautifully illustrates that templating methods involving surfactant-precursor ion pairs allow accessing highly structured surface functionalized silica materials. However, this work exclusively focused on the use of monosilylated co-structure directing agents and the formation of surface functionalized mesoporous silica phases.

Following our work on silica hybrid materials and ionic liquids, we were concerned by the synthesis of silica based materials bearing covalently attached ionic groups. We focused in particular on the formation of materials containing ionic substructures which are similar to ionic liquids or inspired by them [31–33].

**Figure 8.**
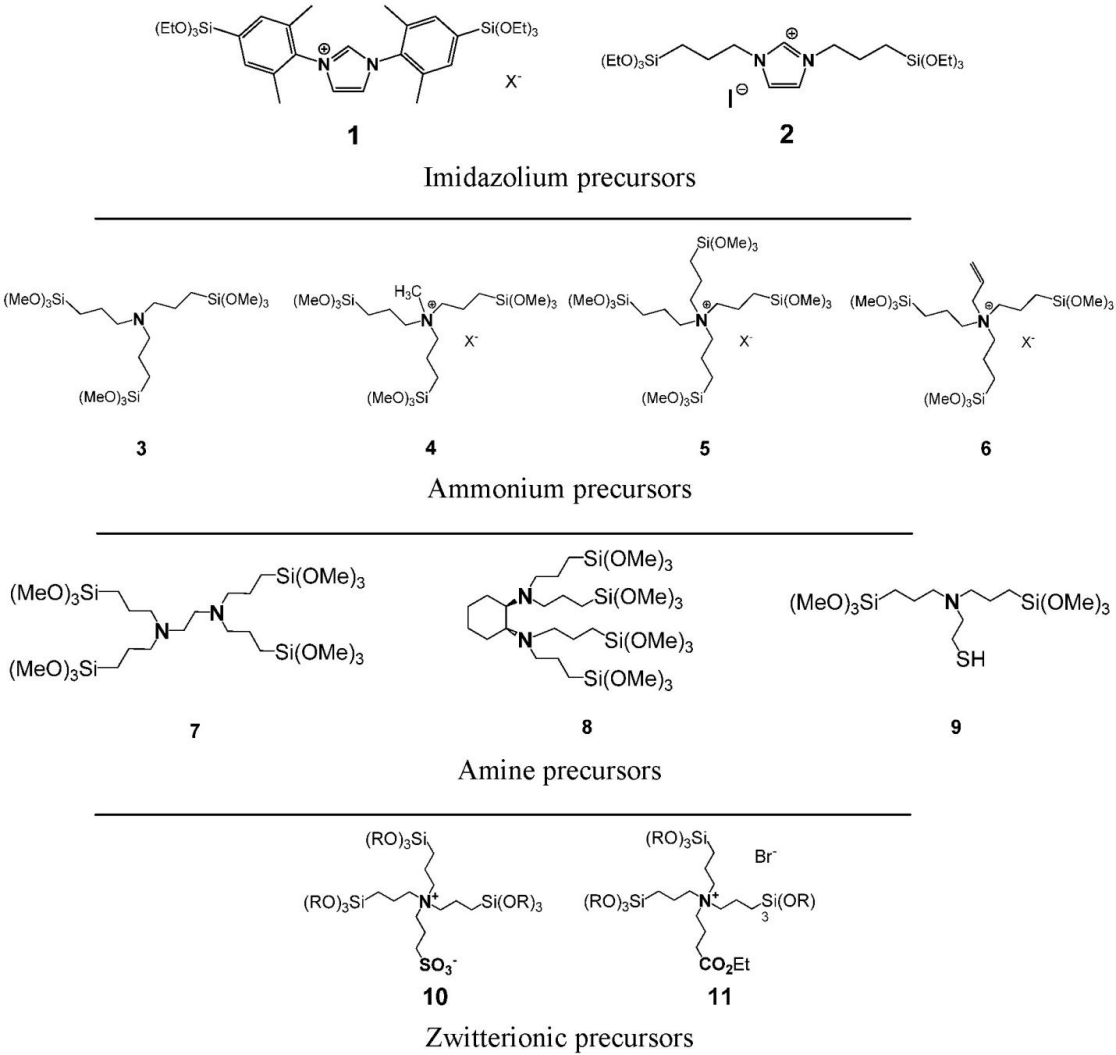
Families of ionosilica precursors studied in template directed hydrolysis-polycondensation reactions: from upper to lower: diaryl- and dialkyl-imidazolium precursors **1** and **2**; ammonium precursors **3**–**6**; functional amine precursors **7**–**9**; zwitterionic precursors **10** and **11**.

In the following, we showed that this synthetic strategy to nanostructured silica based materials is not limited to the formation of surface functionalized silicas, but can also be extended on the preparation of nanostructured silica hybrid materials of the “periodic mesoporous organosilica” type [34–36]. The synthesis of these materials is usually performed via similar synthetic pathways compared to the formation of nanostructured mesoporous silica and involves the use of cationic or non-ionic SDAs [37–39]. The mechanism of formation of nanostructured PMO materials is identical to that of nanostructured silica mesophases and involves ionic interactions or hydrogen bonding between the SDA and hydrolysed organic silylated precursor compounds (Figure 1). We recently showed that the preparation of nanostructured PMO materials can also be achieved from well-defined SDA-precursors ion pairs. In our work, the ionic precursor does not only act as CSDA as described above for the formation of surface functionalized silica, but operates both as structure inducing compound and as molecular brick for solid phase formation at the same time. A large variety of ionic compounds bearing ammonium and imidazolium substructures was used for the formation of these hybrid ionosilica materials (Figure 8).

These compounds include diaryl- and dialkyl-imidazolium precursors, various amine and ammonium halide compounds and ammonium-sulfonate and ammonium carboxylate zwitterions. As differences concerning the behaviour of these compounds appeared in template directed hydrolysis-polycondensation reactions, each type of precursor will be discussed separately in view of the formation of nanostructured ionosilica phases.

### 3.1. Syntheses of Hybrid Ionosilica Mesophases from the Imidazolium Precursors **1** and **2**

In an initial study, we investigated the formation of ionosilica hybrid materials starting from the diaryl- and dialkyl-imidazolium precursors **1** and **2** with the aim to synthesize nanostructured ionosilicas of the “periodic mesoporous organosilica” type. In this context, we were interested in studying a possible influence of the ionic precursors on the architectures of PMO type materials formed in template directed hydrolysis-polycondensation reactions.

**Figure 9.**
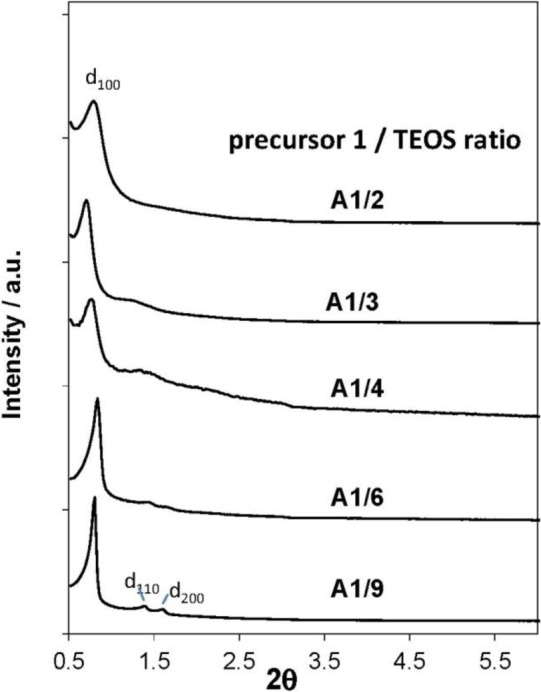
XRD patterns of the materials obtained by hydrolysis/co-condensation reactions from different precursor 1/TEOS mixtures. Reprinted with permission from [40]. Copyright 2009 Royal Society of Chemistry.

The synthesis of silica hybrid materials from the pure di-aryl imidazolium precursor **1** led to the formation of non-structured materials, regardless of the used surfactant [40]. Nanostructured silica hybrid phases could only be obtained via co-condensation reactions from precursor **1**/TEOS mixtures, in particular in the presence of non-ionic SDA such as Pluronic P123. As shown by the XRD patterns of the obtained the materials (Figure 9), the molar ratio between the two co-monomers has deep influence on the morphological regularity of the formed materials: the higher the quantity of TEOS in the hydrolysis-polycondensation mixture, the higher is the degree of order in the formed materials. The highest degree of structural regularity was observed in the case of the material formed from a 1/9 ratio between precursor **1** and TEOS. The formed material displays a typical SBA-15 type architecture with a 2d hexagonal pore arrangement and an average pore diameter of 6 nm.

These results indicate that the presence of the di-aryl-imidazolium precursor in the hydrolysis-polycondensation mixture hampers the formation of nanostructured silica phases. A considerable amount of TEOS in the hydrolysis-polycondensation mixture is necessary to induce the formation of nanostructured phases. A similar behaviour is often observed with classical organic trialkoxysilylated precursors, as the supramolecular organisation of surfactants is strongly affected by the presence of these compounds in the reaction mixture [41–43]. Structured organosilicas hybrid mesophases therefore can only be obtained by limiting the quantity of the organic silylated precursor and the addition of considerable amounts of silica precursors such as TEOS to the reaction mixture. The precursor **1** therefore shows a classical behaviour of conventional organic silica hybrid precursors in template directed hydrolysis-polycondensation reactions.

**Figure 10.**
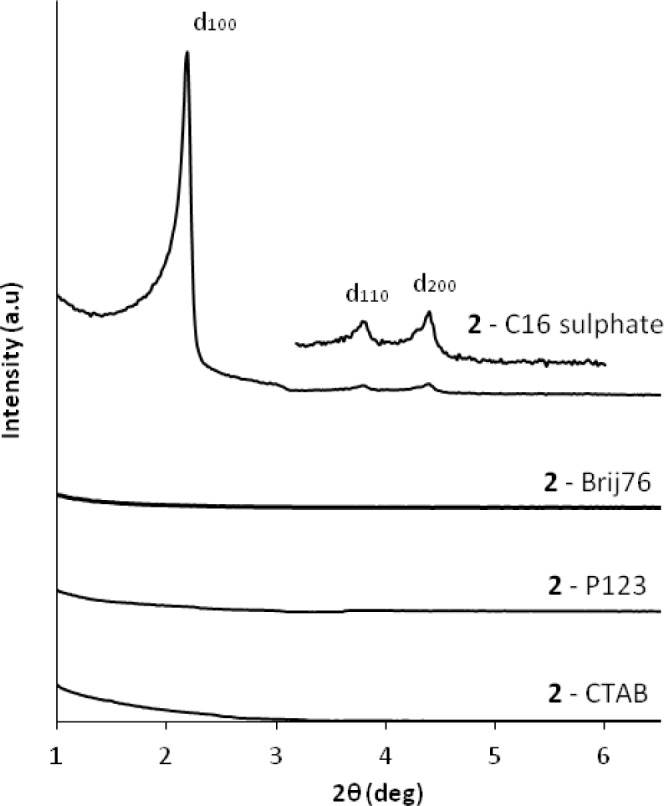
XRD patterns of the as-synthesized materials obtained from precursor **2** in the presence of anionic surfactant C16-sulphate, non-ionic surfactants Brij76 and P123 and cationic surfactant CTAB. Reprinted with permission from [44]. Copyright 2007 Elsevier.

**Figure 11.**
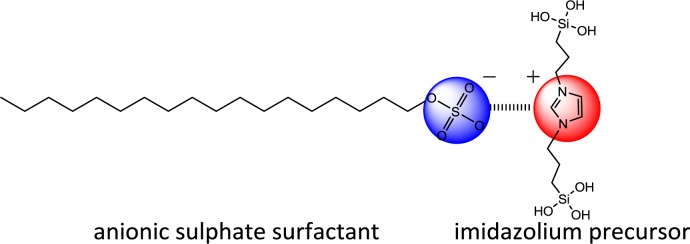
Ionic interactions between anionic SDA and the imidazolium precursor **2**. Reprinted with permission from [44]. Copyright 2007 Elsevier.

Following these results, we studied the use of the related di-alkyl-imidazolium precursor **2** in template directed hydrolysis-polycondensation reactions [44]. Whereas these reactions of this ionic compound in the presence of cationic (CTAB) and non-ionic SDA (Brij76 and P123) led to the formation of non-structured materials, reactions in the presence of anionic surfactants (C16 sulphate) yielded nanostructured materials with 2*d* hexagonal architectures as indicated by the XRD patterns of the materials (Figure 10). This result was surprising as the formation of nanostructured silica hybrid materials in the presence of anionic SDA was never observed before. In fact, the formation of these materials displaying 2d hexagonal phases involves surfactant-precursor interactions as shown in Figure 11, similar to the SDA-CSDA-interactions described by Che *et al*. [18] in the field of the formation of surface functionalized silicas.

**Figure 12.**
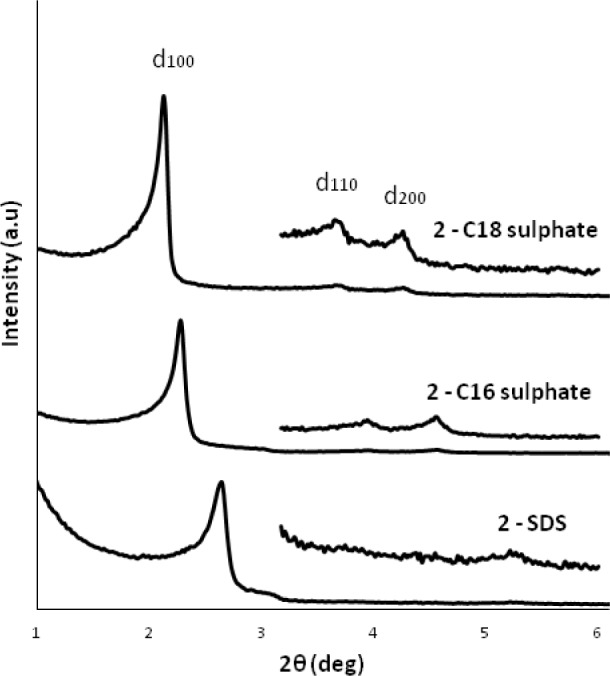
XRD patterns of the as-synthesized materials obtained from precursor **2** in the presence of anionic surfactants C12-sulphate (SDS), C16-sulphate and C18-sulphate. Reprinted with permission from [44]. Copyright 2007 Elsevier.

The templating effect and the ionic interaction between ionic imidazolium precursor and SDA were monitored using surfactants with different alkyl chain length. The XRD patterns of the materials obtained in the presence of dodecyl (C12), hexadecyl (C16) and octadecyl (C18) sulphate anions clearly show a shift of the 1 0 0 reflexion towards bigger distances (C12-sulphate: 3.3 nm, C16-sulphate: 3.9 nm, C18-sulphate: 4.2 nm) thus indicating an increase of the *d*-spacing in these materials which is in direct relationship to the length of the alkyl chain of the anionic surfactant (Figure 12). This tendency clearly shows the imprint of the supramolecular aggregates of the anionic surfactant towards the nano-structuration of these materials. However, it has to be mentioned that all XRD measurements were performed with silica-surfactant nanocomposites, as surfactant elimination by washing yielded amorphous and non-porous materials. This insufficient morphological stability is probably due to a high flexibility on the molecular level, induced by the alkyl chains of the imidazolium precursor.

**Figure 13.**
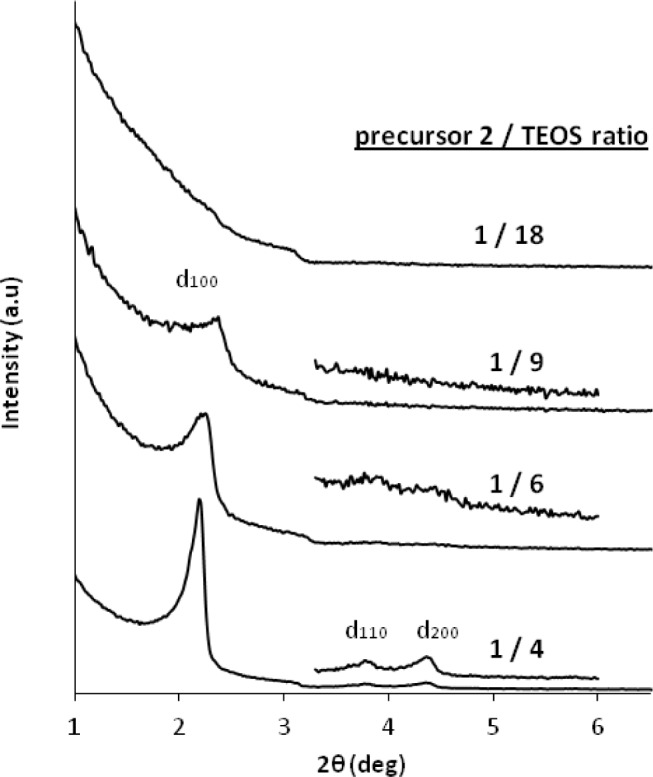
XRD patterns of the as-synthesized materials obtained by co-condensation reactions from different molar precursor **2**/TEOS ratios in the presence of C16-sulphate. Reprinted with permission from [44]. Copyright 2007 Elsevier.

Finally, we studied co-condensation reactions of the ionic imidazolium precursor **2** with TEOS. This study is of particular interest in order to rigidify the material and to access porous hybrid ionosilicas. As observed in the case of the co-condensation reactions involving precursor **1**, the molar precursor/TEOS ratio has deep influence on the formation of silica hybrid mesophases displaying defined architectures. However, using the dialkyl-imidazolium precursor **2** as ionosilica precursor, we observed an inverse tendency with respect to the results obtained with the di-aryl-imidazolium precursor **1**. Using the dialkyl imidazolium precursor **2**, nanostructured materials were only formed from co-monomer mixtures containing a high amount of the ionic precursor. In contrast, the presence of high amounts of TEOS hampers the formation of structured ionosilica hybrid mesophases (Figure 13). This result is in line with the observation that the hydrolysis-polycondensation of TEOS in the presence of anionic surfactant yields amorphous silica materials.

These results illustrate that the imidazolium precursors **1** and **2** show a very different behaviour in template directed hydrolysis-polycondensation reactions despite their very similar molecular structure. Whereas the diaryl-imidazolium precursor **1** shows a rather classical behaviour in these reactions and led to the formation of structured materials only via co-condensation reactions with TEOS, precursor **2** can be used for the synthesis of nanostructured ionosilica materials by template directed hydrolysis-polycondensation reactions in the presence of anionic surfactants. In these reactions, the formation of structured ionosilica mesophases is induced by ionic precursor-surfactant interactions, which is a real particularity of this family of precursors compared to non-ionic compounds.

These differences between the precursors **1** and **2** are probably due to the higher sterical shielding of the imidazolium groups in precursor **1**, whereas the higher morphological flexibility of the alkyl chains ensures efficient precursor-surfactant interactions in the case of precursor **2**.

### 3.2. Syntheses of Hybrid Ionosilica Mesophases from Amine and Ammonium Precursors

In the following, the “anionic templating” strategy was applied to other types of organo-cationic precursors, in particular to the silylated ammonium salts.

A series of new compounds was obtained from the *tris*-trialkoxysilylated amine precursor **3**. These compounds were easily accessible via alkylation reactions. For the first time, we compared the precursors **3**–**5** (Figure 8) in the formation for anionic _emplate silica hybrid materials.

**Figure 14.**
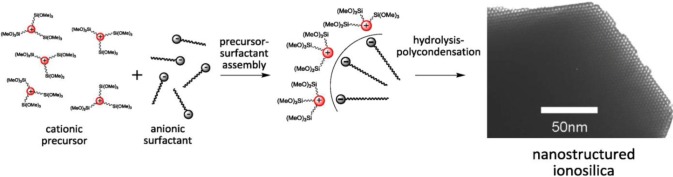
Synthesis of nanostructured ionosilica mesophases by anionic templating from cationic ammonium precursors.

Similarly to the previously described imidazolium salts, hydrolysis-polycondensation reactions involving ammonium salts derived from the neutral amine compound **3** gave rise to the formation of structured materials only in the presence of anionic surfactants. The use of non-ionic or cationic surfactants led to porous but non-structured materials. Once more, this result can be attributed to ionic interactions between the cationic centre of the ammonium precursor and the anionic head-group of the SDA (Figure 14).

**Figure 15.**
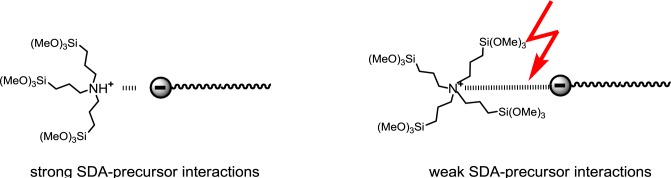
SDA-precursor interactions as a result of steric shielding of the cationic charge of the precursor. Reprinted with permission from [45]. Copyright 2010 Royal Society of Chemistry.

The constitution of the precursor was found to be an important parameter in light of the formation of structured phases. The more the cationic centre of the precursor is sterically shielded, the more the ionic interactions with the anionic SDA are weakened and the less structured are the formed materials (Figure 15). It therefore clearly appears that the formation of structured silica hybrid mesophases is precursor-induced and does not result from siloxy-surfactant interactions.

This method to access nanostructured ionosilica mesophases was found to be generally applicable to various types of functional trialkoxysilylated ammonium precursors [46]. These precursors include compounds containing chelating ethylenediamine groups (precursor **7**), chiral diaminocyclohexane substructures (precursor **8**) or metal-complexing thiol groups (precursor **9**). On the other side, tetraalkyl-ammonium precursors such as the allyl precursor **6** were also studied.

The use of this series of functional amine and ammonium precursors in template directed hydrolysis-polycondensation reactions involving anionic surfactants confirmed the previously described results obtained with the imidazolium compound **2** and ammonium precursors **3**–**5**. The structural regularity depends on the strength of the ionic interactions between the anionic SDA and the precursor. The relatively shielded quaternized ammonium precursors **5** and **6** therefore generally led to materials with low structure regularity and low long-range order. In contrast, hydrolysis polycondensation reactions of the amine precursors **7**–**9** led to highly structured as-synthesized materials (surfactant-ionosilica nanocomposites) displaying 2d hexagonal architectures on a mesoscopic length scale. However, the elimination of the anionic surfactant by washing gave amorphous materials. Similarly, as reported for the imidazolium precursor **1**, the morphological stability of these materials is not sufficient to maintain the regular architecture during surfactant elimination and to form porous and structured ionosilica mesophases without the addition of silica precursors such as TEOS.

**Figure 16.**
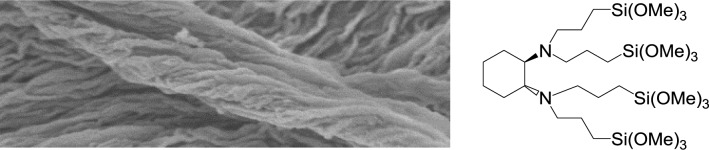
SEM-image of an ionosilica hybrid materials synthesized from the chiral (1*R*,2*R*)-diaminocyclohexane derived precursor **7**. Reprinted with permission from [46]. Copyright 2010 Elsevier.

A special case is the chiral (1*R*,2*R*)-diaminocyclohexane derived precursor **7**. Hydrolysis-polycondensation reactions with this precursor allowed synthesizing a material with fibre-like morphology displaying helicoidal arrangements of bundles of fibres (Figure 16). As already mentioned for the formation of helicoidally twisted rods formed from chiral SDA/non-chiral CSDA ion pairs (*vide supra*), this result shows that the use of non-chiral SDA/chiral precursor also allows transcribing chirality from a supramolecular level to the microscopic length scale of a silica hybrid material.

### 3.3. Zwitterionic Ionosilicas

Following the results on nanostructured ammonium ionosilicas, the use of zwitterionic ammonium sulfonate and ammonium carboxylate precursors **10** and **11** (Figure 8) in template directed hydrolysis-polycondensation reactions was investigated [47]. These precursors show a modified behaviour compared to the previously discussed ammonium precursors due to their zwitterionic nature. The formation of nanostructured materials from these compounds was only observed in the presence of cationic surfactants such as CTAB. We suppose that ionic interactions between the anionic sulfonate group of the zwitterionic precursor and the cationic head-group of CTAB are responsible for the formation of structured phases in this case. This supposition is supported by the fact that hydrolysis-polycondensation reactions starting from conventional ionic precursors such as the trialkoxysilylated allyl-precursor **6** (Figure 8) under identical reactions conditions yielded non-structured materials. This comparison therefore indicates that the formation of structured phases from zwitterionic precursor involves a strong contribution of the anionic sulfonate group.

**Figure 17.**
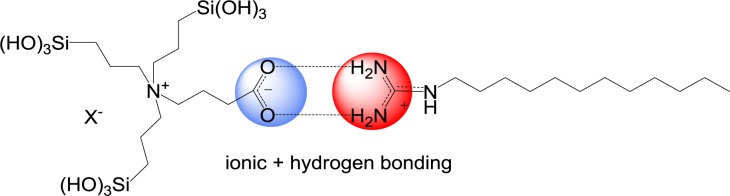
Schematic illustration of ion pairing between guanidinium surfactant and the zwitterionic ammonium carboxylate precursor involving ionic interactions and hydrogen bonding. Reprinted with permission from [48]. Copyright 2012 Wiley-VCH.

The use of a similar ammonium-carboxylate precursor gives slightly different results [48]. Cationic templating approaches with CTAB do not allow accessing nanostructured phases starting from this compound. However, moderately structured materials could be obtained using another cationic SDA, e.g., long-chain substituted guanidinium salts. These surfactants probably have a higher affinity towards the carboxyl group of this specific precursors compared to ammoniums salts such as CTAB (Figure 17). This example highlights that both precursor and surfactant are involved in the formation of structured phases. High affinities between these two compounds are essential in order to form catanionic surfactant and lyotropic phases which can subsequently be transformed into structured ionosilica materials of the PMO type. Therefore, it clearly appears that the formation of surfactant-precursor ion pairs is crucial in view of the formation of structured phases in precursor mediated syntheses of nanostructured ionosilicas.

## 4. Conclusions

Precursor mediated syntheses of structured silica and silica hybrid materials were recently introduced, in particular by the works of Che *et al*. [17–19,21,23,24,26,27,30] and our group [33,40,44–49,50]. This synthetic methodology benefits from the formation of surfactant-precursor ion pairs in the hydrolysis-polycondensation mixture. Contrary to the conventional soft templating methods to mesoporous silicas, this new method involves the organic precursor and therefore opens multiple routes to control the formation of nanostructured silica based mesophases. Key parameters for this technique are, however, the packing parameter of the structure directing agent (SDA) and the affinity between surfactant and precursor.

The universality of the approach has firstly been illustrated in the field of the synthesis of different types of silica mesophases, which are often accessible by judicious changes in the reaction conditions. In this area, the transcription of the chirality of the chiral surfactant to the mesoscopic level and the formation of helicoidally twisted rods of nanostructured silica is certainly the most intuitive example. Furthermore, it has been shown that this approach is not limited on anionic SDA/cationic precursor ion pairs, but can also be extended to the formation of nanostructured silica bearing carboxylate groups or combined amine/carboxylate pairs. Very recently, polyamine/anionic surfactant supramolecular aggregates have also been shown to afford nanostructured silica mesophases [51]. This result highlights the high potential of the rational design ionic soft templates in the field of synthesis of structured silica mesophases via template directed approaches.

Regarding the synthesis of ionosilica hybrid materials of the PMO-type, precursor mediated approaches allow also access to materials displaying particular architectures, which are not accessible via conventional synthetic pathways, *i.e.*, soft-templating hydrolysis-polycondensation reactions in the presence of cationic or non-ionic structure directing agents. For example, the formation of nanostructured organosilicas bearing amine functions is only possible via anionic templating starting from cationic ammonium precursors. Similar to the formation of other types of surface functionalized silica, this approach has also been extended to other types of original materials such as zwitterionic PMOs. Finally, chiral transcription from the molecular to the microscopic level has also been observed with chiral precursors and led to a material with helicoidal morphology.

In conclusion, precursor mediated formation of structured silica based materials appears as a universal and highly flexible method to control the texture of silica materials in particular on a mesoscopic level. This synthetic strategy is highly flexible as both precursor and structure directing agent can be designed. Future developments can be expected in particular at the nanostructured silica/poly(ionic liquid) interface as already mentioned in the field of anionic surfactant/polyamine aggregates for soft templating approaches [51]. Precursor mediated synthesis of nanostructured silica mesophases is therefore an interesting and complementary alternative for the synthesis of mesoporous silica and silica hybrid phases. As mostly ionic precursors are used in order to increase the affinity towards the SDA, this strategy opens the route to a multitude of interesting functional and structured ionosilicas. Besides the investigation of the formation of tailor-made silica mesophases, the resulting ionosilicas are highly promising functional organosilicas. These materials have great potential for applications in catalysis, sensing and separation.
